# Innate lymphoid cells and tumor-derived lactic acid: novel contenders in an enduring game

**DOI:** 10.3389/fimmu.2023.1236301

**Published:** 2023-10-05

**Authors:** Mateusz Marciniak, Marek Wagner

**Affiliations:** ^1^ Cancer Biomarkers Research Group, Łukasiewicz Research Network - PORT Polish Center for Technology Development, Wrocław, Poland; ^2^ Department of Biomedicine, University of Bergen, Bergen, Norway

**Keywords:** innate lymphoid cells, skin, skin cancer, melanoma, lactic acid, lactate, metabolism

## Abstract

Aerobic glycolysis, also known as the Warburg effect, has for a prolonged period of time been perceived as a defining feature of tumor metabolism. The redirection of glucose utilization towards increased production of lactate by cancer cells enables their rapid proliferation, unceasing growth, and longevity. At the same time, it serves as a significant contributor to acidification of the tumor microenvironment, which, in turn, imposes substantial constraints on infiltrating immune cells. Here, we delve into the influence of tumor-derived lactic acid on innate lymphoid cells (ILCs) and discuss potential therapeutic approaches. Given the abundance of ILCs in barrier tissues such as the skin, we provide insights aimed at translating this knowledge into therapies that may specifically target skin cancer.

## Introduction

Lactic acid was identified in 1780 by Carl Wilhelm Scheele who isolated it from sour milk and based on its origin coined the name “Mjölksyra” or “acid of milk” (1). In aqueous solutions with a physiological pH, the hydrophobic acid converts almost entirely into its conjugate base - lactate. In 1856, Louis Pasteur rediscovered lactate as a fermentation product generated by microorganisms (2). While microorganisms also produce other fermentation metabolites, lactate stands out as the predominant product of fermentation in mammals (3). The production of lactate escalates when the demand for ATP and oxygen surpasses the available supply, which commonly happens during periods of intense exercise (4).

Lactate is produced through the transformation of pyruvate, the end product of glycolysis, by the enzyme lactate dehydrogenase (LDH). Under normal aerobic conditions, pyruvate drives ATP production by oxidative phosphorylation. However, when oxygen availability is limited, pyruvate is converted to lactate as a means to replenish NAD^+^ and sustain glycolysis. Interestingly, even in the presence of sufficient oxygen, certain prokaryotic and eukaryotic cells choose to convert pyruvate into lactate. Otto Warburg initially described this metabolic process (known as the eponymous Warburg effect) in tumor cells, which produced lactate and released it extracellularly (5, 6). Several factors drive the Warburg effect, including the activation of growth factor signaling pathways driven by oncogenes. For example, the PI3K/AKT/mTOR pathway increases glucose uptake and glycolysis in cancer cells (7). The upregulation of glucose transporters, such as GLUT1, facilitates the efficient uptake of glucose. On the other hand, monocarboxylate transporters (MCTs), specifically MCT1 and MCT4, facilitate the release of lactate into the extracellular space (8). These transporters enable the simultaneous movement of monocarboxylate ions such as lactate and protons across the plasma membrane, allowing bidirectional transport. While systemic lactate concentrations are tightly regulated at approximately 1-2 mM, certain conditions including cancer can result in significantly higher levels (9, 10). In addition, the concentrations of lactate beyond physiological ranges have the potential to impact cellular function. For example, lactic acid has been found to inhibit the differentiation of human dendritic cells (DCs) (11). Furthermore, lactic acid suppresses the production of cytokines by T cells and impedes their proliferation (12). Tumor lactic acidosis also restrains the tumor immunosurveillance carried out by T cells (13). In contrast, macrophages, following the stimulation with lactic acid, acquire a protumorigenic alternatively activated phenotype (14).

Innate lymphoid cells (ILCs) represent the first line of defense. However, the precise impact of tumor-derived lactic acid on ILCs is incompletely understood. In this regard, we present a summary of recent findings regarding the influence of lactic acid on ILCs and examine potential therapeutic approaches. We place particular emphasis on the relevance of ILCs in skin cancer, considering their abundance in barrier tissues such as the skin (15).

## ILCs and melanoma

Innate lymphoid cells (ILC) comprise a family of recently discovered lymphocytes, which exhibit multifaceted functions. Based on the expression of distinctive cytokines and transcription factors they have been categorized into five subsets, namely natural killer (NK) cells, group 1 ILCs (ILC1s), ILC2s, ILC3s, and lymphoid tissue inducer (LTi) cells (16). NK cells differentiate with the assistance of the transcription factor eomesodermin (Eomes) and produce cytotoxic mediators such as perforin and granzymes in mice, while in humans they can also produce granulysin (17, 18). ILC1s are regulated by the T-box transcription factor T-bet independently of Eomes and produce interferon (IFN)-γ (18). ILC2s, governed by the transcription factor GATA binding protein 3 (GATA3) generate type 2 cytokines, including interleukin (IL)-4, IL-5, and IL-13 (18, 19). ILC3s, on the other hand, rely on the transcription factor RAR-related orphan receptor gamma t (RORγt) and produce cytokines such as IL-17A and IL-22. It is important to note, however, that ILC progenitors (ILCPs) represent ILC3s in human peripheral blood, which indicates their lack of maturity (18, 20). Last in order, LTi cells, which contribute to the development of lymphoid tissues during fetal stages, produce lymphotoxin (LT), a member of the TNF cytokine family (18).

Emerging body of evidence underscores the influence of environmental stimuli on the function of ILCs (15). ILCs possess receptors that allow them to survey the surroundings and mount responses against threats to tissue integrity. Rather than relying on antigens like T and B cells, ILCs swiftly respond to stress signals such as an array of cytokines released by epithelial and myeloid cells (15). ILC1s, similar to NK cells, rely on IL-15 during their development. Additionally, IL-12, IL-18, and IL-15 serve as activators for both ILC1s and NK cells (18, 21). Monocytes and activated DCs contribute to the secretion of IL-12 and IL-18, whereas activated monocytes, macrophages, and various non-hematopoietic cells, including epithelial and fibroblast cell lines produce IL-15 (14). On the other hand, ILC2s predominantly respond to IL-33, IL-25, and thymic stromal lymphopoietin (TSLP), either individually or in conjunction with IL-33 (19). These cytokines stem from diverse cell types such as epithelial and endothelial cells, smooth muscle cells, fibroblasts, macrophages, and activated DCs (18). IL-25 production arises from activated Th2 cells, macrophages, eosinophils, basophils, mast cells, tuft cells, as well as fibroblasts, epithelial and endothelial cells. Meanwhile, TSLP expression primarily characterizes skin epithelial cells (18). Finally, the activation of ILC3s and LTi cells hinges on IL-1β and IL-23, both of which are generated by activated DCs and macrophages (18).

ILCs predominantly reside in barrier tissues. While the conventional NK (cNK) cells are primarily found circulating in the blood, specific non-lymphoid tissues, including the skin, also harbor subsets of tissue-resident NK (trNK) cells (22). Whereas cNK cells are positive for T-bet and Eomes, trNK cells are negative for Eomes but express T-bet (23). The participation of NK cells in antitumor immunity is undeniable. Their prevalence in the bloodstream correlates with reduced metastatic potential in various human cancers, including melanoma (24, 25). However, our comprehension of the role and function of the remaining ILC subsets in skin malignancies is still at an early stage. Further confounding the issue is the plastic potential of ILCs (26, 27). However, recent findings suggest that ILCs play a significant role in the regulation of melanoma, the most aggressive form of skin cancer traditionally associated with immune responses primarily mediated by adaptive immunity (reviewed in (15)).

Melanoma develops from melanocytes, which are found in the skin (i.e. the basal layer of the epidermis), eyes (i.e. the uveal tract) and hair (i.e. the hair follicle). Although it is less common than basal cell carcinoma (BCC) and squamous cell carcinoma (SCC) it is responsible for the majority of deaths related to skin cancer, partially due to its capacity to metastasize to distant organs. Aerobic glycolysis plays a crucial role in providing the necessary metabolic energy for melanoma cells to rapidly proliferate and metastasize. Indeed, the expression of *LDHA*, which encodes a subunit of LDH, correlates with shorter overall survival in metastatic melanoma patients (13). This highlights the significance of the association between the production of lactic acid and patient survival.

## Lactic acid and ILCs

**Figure 1:**
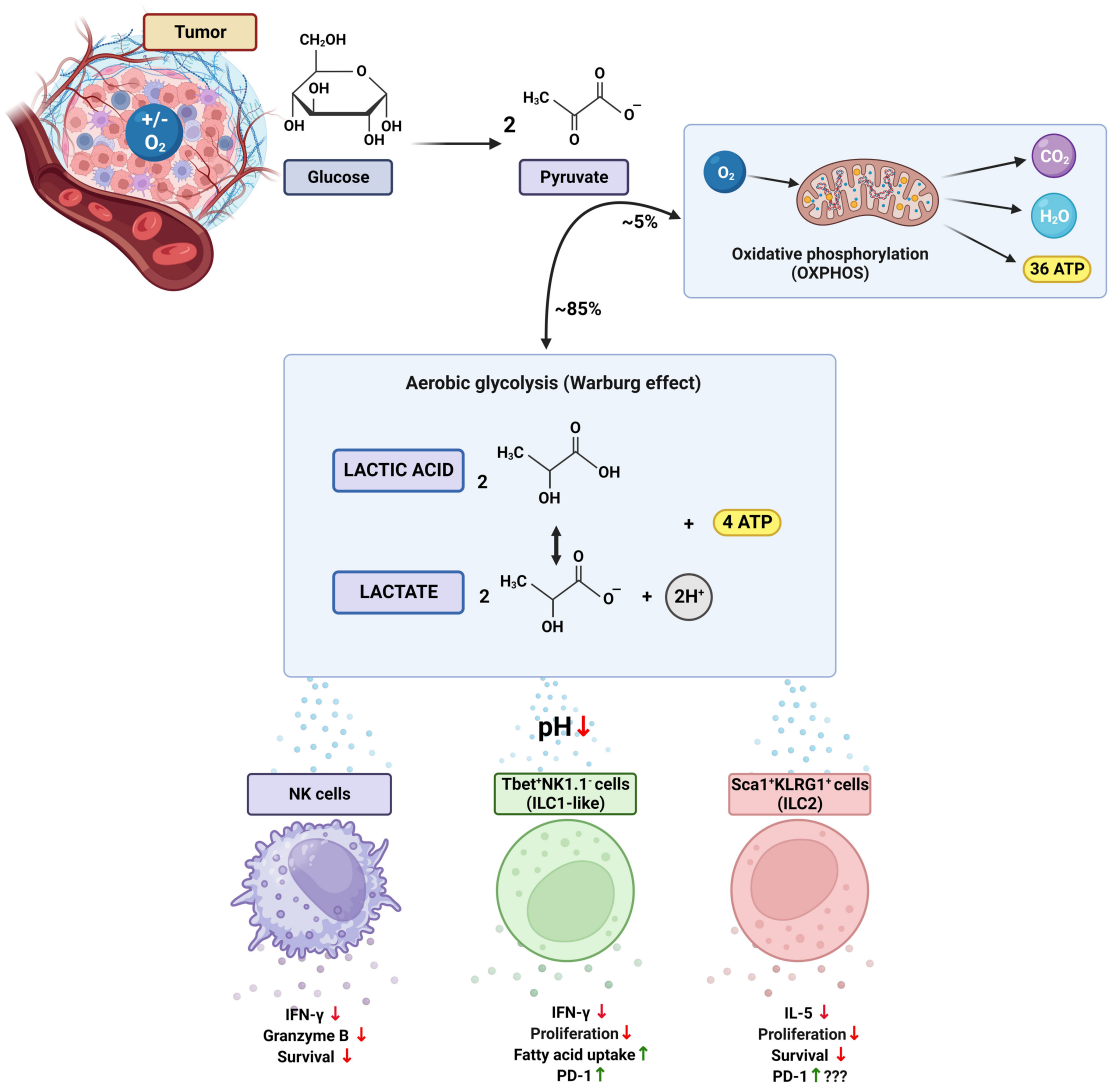
The Warburg effect and its impact on ILCs in the tumor microenvironment. The Warburg effect (or aerobic glycolysis) is characterized by a metabolic reprogramming in which cancer cells rely on glycolysis regardless of the availability of oxygen. In this process, most of the pyruvate generated from glucose undergoes glycolytic transformation, resulting in the production of lactate, protons, and ATP. Lactate and lactic acid contribute to acidification of the tumor environment, thereby disrupting the function of immune cells, including innate lymphoid cells (ILCs). Consequently, NK cells show reduced production of INF-γ and granzyme B, as well as significantly diminished survival. In T-bet^+^NK1.1^-^ ILC1-like cells, inhibition of proliferation and INF-γ secretion occurs accompanied by an increase in PD-1 expression and uptake of fatty acids. Negative effects are also observed in Sca1^+^KLRG1^+^ ILC2 cells, including impaired proliferation, decreased secretion of IL-5, and reduced survival. The impact of elevated PD-1 expression in ILC2s has yet to be fully understood. T-bet, T-box transcription factor; Sca1, stem cell antigen-1; KLRG1, killer cell lectin-like receptor subfamily G member 1; INF-γ, interferon gamma; PD-1, programmed cell death receptor 1. Figure created with BioRender.com.

Melanoma cells convert up to 80% of glucose to lactate (28, 29). An increased production of lactate and thus an increased acidity of the tumor microenvironment contribute to the mechanism of tumor escape from immunosurveillance mediated by cells of the immune system, including ILCs (30, 31). Indeed, it has been revealed that lowering the pH from 6.8 to 6.0 leads to a significant decrease in the cytotoxic activity of mouse NK cells as reflected by lowered mRNA levels of granzyme B and perforin. In addition, exposure of human NK cells to lactate decreases the expression of the NKp46 activation receptor. Interestingly, the inhibitory effect of lactate on the expression of natural cytotoxicity receptors has been considered gene-specific, as there was no significant change in the level of NKp30, NKp44, and NKG2D (32). Using mouse melanoma as a model, NK cells exhibited higher expression of IFN-γ and granzyme B in tumors with reduced lactic acid production compared to control tumors (**Figure 1**). Furthermore, lactic acid concentrations exceeding 20 mM induced apoptosis in NK cells *in vitro*, which might help explain the smaller proportion of NK cells observed in tumors with higher lactate concentrations (13). Although not studied in the context of cancer, hepatic trNK cells, in contrast to cNK cells, have been found to undergo rapid apoptosis during murine cytomegalovirus (MCMV) infection as a consequence of an increased sensitivity to lactic acid, which highlights the distinctive properties of the tissue-resident population of NK cells (33). It remains to be determined whether tissue-resident NK cells in the skin exhibit the same heightened responsiveness to lactic acid as observed in the liver.

The acidification induced by lactic acid has also been found to inhibit the activity of the transcription factor, nuclear factor of activated T cells (NFAT), leading to decreased production of IFN-γ (13). This direct connection between lactate levels and NFAT activity is significant since NFAT is responsible for coordinating various activities not only in T cells but also in other immune cell types, including ILCs (23). Indeed, recent studies have demonstrated that leukotriene receptors activate NFAT in ILC2s (34). ILC2s were first identified in adipose tissue and implicated in the induction of an anti-inflammatory response (35). Recently, increased lactate production by adipocytes has been found to promote adipose tissue macrophage polarization to an inflammatory state in the context of obesity (36, 37). Considering the notion that some tumors grow in close proximity to adipose tissue (e.g. melanoma) or spread to lymph nodes, which are typically shrouded by adipocytes, the impact of lactate production by adipocytes on ILC2s and its role on tumor growth remains to be assessed (38–40). Nevertheless, ILC2s exposed *in vitro* to lactate have been characterized by an inhibited cytokine production, suppressed proliferation and decreased survival (**Figure 1**). Importantly, ILC2s more effectively controlled the growth of melanomas with reduced lactic acid production when compared to control tumors following the treatment with IL-33 (41). It has also been revealed that the blockade of programmed cell death protein 1 (PD1) further enhances the tumoricidal capacity of ILC2s induced by IL-33. In one study, PD1 inhibition increased TNF-α production by ILC2s, leading to direct inhibition of metastatic spread of intravenously administered melanoma cells (42). Another study found that the blockade of PD1 enhanced the tumoricidal potential of ILC2s specifically through the recruitment of eosinophils via granulocyte-macrophage colony-stimulating factor (GM-CSF) (43). The impact of lactic acid on the expression of PD1 on ILC2s is yet to be determined, although it has been shown that PD1 blockade is less effective in highly glycolytic tumors (44). Nevertheless, tumor-derived lactate has been found to enhance the expression of PD1 on a subset of ILCs that are T-bet^+^NK1.1^−^ within the tumor microenvironment (**Figure 1**), which led to diminished signaling of mammalian target of rapamycin (mTOR) together with elevated uptake of fatty acids. Consistent with the metabolic alterations, PD1-deficient T-bet^+^NK1.1^−^ ILCs have been characterized by an increased expression of IFN-γ and granzyme B and K. In addition, the presence of PD1-deficient T-bet^+^NK1.1^−^ ILCs has been associated with inhibited growth of melanomas in mice (45). Although further studies are necessary to fully understand the impact of tumor-derived lactate on ILCs, these findings pave the way for exploring strategies aimed at regulating the lactate levels within the tumor microenvironment.

Given the metabolic heterogeneity observed in solid tumors, it should be noted, however, that cancer cells have also been found to utilize lactate alongside glucose as a carbon source (46). Additionally, metabolic interactions between cancer cells and stromal cells have been observed. For example, cancer-associated fibroblasts (CAFs) have been found to undergo aerobic glycolysis, leading to lactate production, which has been subsequently taken up and utilized by cancer cells to meet their energy requirements. This phenomenon has been referred to as the “reverse Warburg effect” (47).

## Potential therapeutic approaches

The effectiveness of immunotherapies is often affected by the metabolic reprogramming, which shapes the diversity of immune cells that infiltrate the tumor microenvironment (48). The correlation between an increased “prior to treatment” expression level of LDH and poor outcome has been observed in melanoma patients receiving immune checkpoint inhibitors targeting PD1 such as Nivolumab or Pembrolizumab (49–51). Therefore, the possibility to regulate the lactate levels within the tumor microenvironment will be crucial in harnessing the power of ILCs to treat a variety of cancers, including melanoma.

One way to reduce the lactate levels in the tumor microenvironment involves targeting enzymes responsible for lactate production. LDH is known to facilitate the reversible transformation of pyruvate into lactate (48). Active LDH represents either homo- or heterotetrameric structure generated through the association of distinct subunits, namely M and H, which are encoded by specific genetic entities known as *LDHA* (M) and *LDHB* (H), respectively. The nomenclature for these subunits was assigned owing to their initial identification in the muscle (M) and heart (H) tissue. The tetrameric structure of LDH gives rise to five specific isoenzymes, demonstrating variations in the relative abundance of LDHA and LDHB subunits as well as their distribution across diverse tissues (52, 53). While various studies have revealed that the suppression of *LDHA* gene expression cripples tumor cell proliferation both *in vitro* and *in vivo* (54–57), it has also been suggested that the complete inhibition of the tumor growth can only be achieved through simultaneous disruption of both *LDHA* and *LDHB* genes. For example, B16F10 melanoma cells were still able to secrete substantial amounts of lactate following the elimination of either LDHA or LDHB alone. Melanoma cells, however, stopped proliferating under hypoxic conditions following the simultaneous elimination of both LDHA and LDHB. In contrast, the cells were able to grow under normoxic conditions by reactivating oxidative phosphorylation, which resulted in a twofold decrease in the proliferation rate compared to control cells. Additionally, GNE-140, which targets both LDHA and LDHB, was sufficient to mimic the effect of the simultaneous elimination of both isoforms in melanoma cells in terms of inhibition of glycolysis and reactivation of oxidative phosphorylation in WT cells (52).

Another strategy to reduce lactic acid levels in the tumor microenvironment involves lactate oxidation to pyruvate with the formation of hydrogen peroxide (H_2_O_2_) catalyzed by lactate oxidase (LOX) (58). In this scenario, LOX is encapsulated using cationic polyethyleneimine (PEI) and copper ions (Cu^2+^). The cationic PEI component actively traps lactate, which is then degraded by an encapsulated reservoir of LOX. In addition, Cu^2+^ ions serve as a catalyst in the Fenton reaction, which decomposes H_2_O_2_ into cytotoxic hydroxyl radicals (·OH) and alkalizing hydroxyl anions (OH^-^). Importantly, excessive reactive oxygen species (ROS) generated in the reaction have been found to induce immunogenic cell death (59).

Alternative method to regulate the lactate levels in the tumor microenvironment involves the use of synthetic D-lactate dimers (60). Human cells predominantly produce L-lactate, which is precipitated by D-lactate polymers, stereoisomers commonly produced by the gut microbiome (61). It has previously been reported that synthesized D-lactate polymers formed stereocomplexes with L-lactate, leading to the depletion of plasma levels of L-lactate. Importantly, D-lactate dimers (DLADs) have also demonstrated toxicity towards human melanoma cells *in vitro* (60, 62). Moreover, the intratumoral administration of DLAD has been found to inhibit the growth of human melanomas in immunodeficient mice (60).

Transmembrane MCTs serve as another important target. MCTs, play a key role in governing the lactate levels in the tumor microenvironment. MCTs facilitate the efflux of lactate and protons to the extracellular environment causing acidification of the tumor milieu (63, 64). Inhibition of MCT-1 in melanoma cells has been linked to decreased metastatic potential of mouse and human melanomas. Additionally, it has been observed that the inhibition of MCT-1 or MCT-4 induces oxidative stress through the suppression of lactate export and reduction of glycolysis (65).

The inhibition of proteins responsible for distribution of ions within the tumor microenvironment such as proton transporters (i.e. carbonic anhydrase (CA)) and vacuolar-type membrane-embedded protein complexes that operate as ATP hydrolysis-driven proton pumps (V-ATPase) serve as a means to neutralize acidic pH (66–68). An increased expression of carbonic anhydrase IX (CAIX) has been found in mouse melanoma cells cultured in acidified medium (pH 6.7 ± 0.1) when compared to standard conditions (pH 7.4 ± 0.1) (69). The inhibition of CAIX, using small molecule inhibitor SLC-0011, resulted in the suppression of the extracellular acidification. Importantly, the inhibition of CAIX in combination with immune-checkpoint inhibitors has been found to enhance the response to anti-PD-1 and anti-CTLA-4 therapies as revealed using a mouse model of melanoma (70). The suppression of V-ATPases in mouse and human melanoma cells, on the other hand, has been achieved through the utilization of Myrtenal, a monoterpene derived from plants. It has been found that Myrtenal perturbed the electrochemical proton (H^+^) gradient across the cellular membranes and induced apoptosis. In addition, it has significantly attenuated the migratory and invasive capacities of tumor cells *in vitro* and *in vivo* (71).

The impact of a commercially available alkalizing agents such as Basenpulver^®^ (BP) on tumor growth has also been assessed using a mouse model of melanoma. Initially, it has been revealed that the administration of BP significantly inhibited the proliferation of mouse and human melanoma cells *in vitro*. *In vivo*, significantly slower growth of melanomas has been observed following prolonged BP supplementation of mice (72). Although more research is needed, results from this study provide evidence that targeting the pH of the tumor microenvironment might be achieved through the systemic approach.

However, it is crucial to understand the limitations of the aforementioned therapeutic strategies. The utilization of lactate as a therapeutic target in clinical practice, to date, has been sporadic (reviewed in (73)). Bluntly interfering with glycolysis, with the aim of reducing lactic acid production in tumor cells, can inadvertently harm normal cells and tissues, resulting in potential toxicities. Tumor cells may also develop resistance to inhibitors of glycolysis, fostering alternative metabolic adaptations that could be more aggressive and difficult to treat. Therefore, it is paramount to meticulously weigh the benefits of targeting lactic acid production against the potential adverse effects caused by meddling with vital cellular processes.

## Discussion

The metabolic reprogramming enables cancer cells to meet the demands of rapid tumor growth and progression. An increased glycolysis leads to the accumulation of lactic acid in the tumor microenvironment. This phenomenon can also modulate the function of ILCs, ultimately affecting their response against cancer. The specific interactions between lactate and ILCs have only recently garnered attention. Several important questions remain unanswered thus providing avenues for future research. Firstly, what are the specific mechanisms by which lactic acid affects the phenotypic and functional properties of ILCs, including their cytotoxic capabilities? Secondly, how does lactic acidosis impact the metabolic reprogramming of ILCs? Moreover, how does lactic acidosis influence the crosstalk between ILCs and other immune cell populations within the tumor microenvironment? Further research is needed to understand these intricate relationships and determine the context-dependent effects of lactic acidosis on tumor progression and function of immune cells within the tumor microenvironment. These studies will aid in developing targeted therapeutic strategies that exploit the metabolic vulnerabilities of tumors and optimize immune responses for effective cancer treatment.

## Author contributions

All authors listed have made a substantial, direct, and intellectual contribution to the work, and approved it for publication.

## References

[1] Ferguson BS Rogatzki MJ Goodwin ML Kane DA Rightmire Z Gladden LB. Lactate metabolism: historical context, prior misinterpretations, and current understanding. Eur J Appl Physiol (2018) 118(4):691–728. doi: 10.1007/s00421-017-3795-6 29322250

[2] Parks SK Mueller-Klieser W Pouyssegur J. Lactate and acidity in the cancer microenvironment. Annu Rev Canc Biol (2020) 4:141–58. doi: 10.1146/annurev-cancerbio-030419-033556

[3] Psychogios N Hau DD Peng J Guo AC Mandal R Bouatra S. The human serum metabolome. PloS One (2011) 6(2). doi: 10.1371/journal.pone.0016957 PMC3040193 21359215

[4] Goodwin ML Harris JE Hernandez A Gladden LB. Blood lactate measurements and analysis during exercise: a guide for clinicians. J Diabetes Sci Technol (2007) 1(4):558–69. doi: 10.1177/193229680700100414 PMC2769631 19885119

[5] Warburg O. On the origin of cancer cells. Science (1956) 123(3191):309–14. doi: 10.1126/science.123.3191.309 13298683

[6] Vander Heiden MG Cantley LC Thompson CB. Understanding the Warburg effect: the metabolic requirements of cell proliferation. Science (2009) 324(5930):1029–33. doi: 10.1126/science.1160809 PMC2849637 19460998

[7] Hoxhaj G Manning BD. The PI3K-AKT network at the interface of oncogenic signalling and cancer metabolism. Nat Rev Cancer (2020) 20(2):74–88. doi: 10.1038/s41568-019-0216-7 31686003 PMC7314312

[8] Payen VL Mina E Van Hee VF Porporato PE Sonveaux P. Monocarboxylate transporters in cancer. Mol Metab (2020) 33:48–66. doi: 10.1016/j.molmet.2019.07.006 31395464 PMC7056923

[9] Heikenfeld J Jajack A Feldman B Granger SW Gaitonde S Begtrup G. Accessing analytes in biofluids for peripheral biochemical monitoring. Nat Biotechnol (2019) 37(4):407–19. doi: 10.1038/s41587-019-0040-3 30804536

[10] Wagner M Wiig H. Tumor interstitial fluid formation, characterization, and clinical implications. Front Oncol (2015) 5:115. doi: 10.3389/fonc.2015.00115 26075182 PMC4443729

[11] Gottfried E Kunz-Schughart LA Ebner S Mueller-Klieser W Hoves S Andreesen R. Tumor-derived lactic acid modulates dendritic cell activation and antigen expression. Blood (2006) 107(5):2013–21. doi: 10.1182/blood-2005-05-1795 16278308

[12] Fischer K Hoffmann P Voelkl S Meidenbauer N Ammer J Edinger M. Inhibitory effect of tumor cell-derived lactic acid on human T cells. Blood (2007) 109(9):3812–9. doi: 10.1182/blood-2006-07-035972 17255361

[13] Brand A Singer K Koehl GE Kolitzus M Schoenhammer G Thiel A. LDHA-associated lactic acid production blunts tumor immunosurveillance by T and NK cells. Cell Metab (2016) 24(5):657–71. doi: 10.1016/j.cmet.2016.08.011 27641098

[14] Colegio OR Chu NQ Szabo AL Chu T Rhebergen AM Jairam V. Functional polarization of tumour-associated macrophages by tumour-derived lactic acid. Nature (2014) 513(7519):559–63. doi: 10.1038/nature13490 PMC4301845 25043024

[15] Wagner M Koyasu S. Innate lymphoid cells in skin homeostasis and Malignancy. Front Immunol (2021) 12:758522. doi: 10.3389/fimmu.2021.758522 34691082 PMC8531516

[16] Vivier E Artis D Colonna M Diefenbach A Di Santo JP Eberl G. Innate lymphoid cells: 10 years on. Cell (2018) 174(5):1054–66. doi: 10.1016/j.cell.2018.07.017 30142344

[17] Lu CC Wu TS Hsu YJ Chang CJ Lin CS Chia JH. NK cells kill mycobacteria directly by releasing perforin and granulysin. J Leukocyte Biol (2014) 96(6):1119–29. doi: 10.1189/jlb.4A0713-363RR 25139289

[18] Nagasawa M Spits H Ros XR. Innate lymphoid cells (ILCs): cytokine hubs regulating immunity and tissue homeostasis. Cold Spring Harb Perspect Biol (2018) 10(12). doi: 10.1101/cshperspect.a030304 PMC6280706 29229782

[19] Moro K Yamada T Tanabe M Takeuchi T Ikawa T Kawamoto H. Innate production of T(H)2 cytokines by adipose tissue-associated c-Kit(+)Sca-1(+) lymphoid cells. Nature (2010) 463(7280):540–4. doi: 10.1038/nature08636 20023630

[20] Nagasawa M Germar K Blom B Spits H. Human CD5(+) innate lymphoid cells are functionally immature and their development from CD34(+) progenitor cells is regulated by id2. Front Immunol (2017) 8. doi: 10.3389/fimmu.2017.01047 PMC5583608 28912776

[21] Fuchs A Vermi W Lee JS Lonardi S Gilfillan S Newberry RD. Intraepithelial type 1 innate lymphoid cells are a unique subset of IL-12- and IL-15-responsive IFN-gamma-producing cells. Immunity (2013) 38(4):769–81. doi: 10.1016/j.immuni.2013.02.010 PMC3634355 23453631

[22] Sojka DK Plougastel-Douglas B Yang LP Pak-Wittel MA Artyomov MN Ivanova Y. Tissue-resident natural killer (NK) cells are cell lineages distinct from thymic and conventional splenic NK cells. Elife (2014) 3. doi: 10.7554/eLife.01659 PMC3975579 24714492

[23] Daussy C Faure F Mayol K Viel S Gasteiger G Charrier E. T-bet and Eomes instruct the development of two distinct natural killer cell lineages in the liver and in the bone marrow. J Exp Med (2014) 211(3):563–77. doi: 10.1084/jem.20131560 PMC3949572 24516120

[24] Tarazona R Duran E Solana R. Natural killer cell recognition of melanoma: new clues for a more effective immunotherapy. Front Immunol (2015) 6:649. doi: 10.3389/fimmu.2015.00649 26779186 PMC4703774

[25] Wolf NK Kissiov DU Raulet DH. Roles of natural killer cells in immunity to cancer, and applications to immunotherapy. Nat Rev Immunol (2023) 23(2):90–105. doi: 10.1038/s41577-022-00732-1 35637393

[26] Wagner M Moro K Koyasu S. Plastic heterogeneity of innate lymphoid cells in cancer. Trends Cancer (2017) 3(5):326–35. doi: 10.1016/j.trecan.2017.03.008 28718410

[27] Bald T Wagner M Gao Y Koyasu S Smyth MJ. Hide and seek: Plasticity of innate lymphoid cells in cancer. Semin Immunol (2019) 41:101273. doi: 10.1016/j.smim.2019.04.001 30979591

[28] Ratnikov BI Scott DA Osterman AL Smith JW Ronai ZA. Metabolic rewiring in melanoma. Oncogene (2017) 36(2):147–57. doi: 10.1038/onc.2016.198 PMC5140782 27270434

[29] Avagliano A Fiume G Pelagalli A Sanita G Ruocco MR Montagnani S. Metabolic plasticity of melanoma cells and their crosstalk with tumor microenvironment. Front Oncol (2020) 10. doi: 10.3389/fonc.2020.00722 PMC7256186 32528879

[30] Wagner M Koyasu S. Cancer immunoediting by innate lymphoid cells. Trends Immunol (2019) 40(5):415–30. doi: 10.1016/j.it.2019.03.004 30992189

[31] Wagner M Koyasu S. Cancer immunosurveillance by ILC2s. Trends Cancer (2022) 8(10):792–4. doi: 10.1016/j.trecan.2022.06.010 35871054

[32] Husain Z Huang Y Seth P Sukhatme VP. Tumor-derived lactate modifies antitumor immune response: effect on myeloid-derived suppressor cells and NK cells. J Immunol (2013) 191(3):1486–95. doi: 10.4049/jimmunol.1202702 23817426

[33] Dodard G Tata A Erick TK Jaime D Miah SMS Quatrini L. Inflammation-induced lactate leads to rapid loss of hepatic tissue-resident NK cells. Cell Rep (2020) 32(1):107855. doi: 10.1016/j.celrep.2020.107855 32640221 PMC7383148

[34] von Moltke J O'Leary CE Barrett NA Kanaoka Y Austen KF Locksley RM. Leukotrienes provide an NFAT-dependent signal that synergizes with IL-33 to activate ILC2s. J Exp Med (2017) 214(1):27–37. doi: 10.1084/jem.20161274 28011865 PMC5206504

[35] Misawa T Wagner M Koyasu S. ILC2s and adipose tissue homeostasis: progress to date and the road ahead. Front Immunol (2022) 13:876029. doi: 10.3389/fimmu.2022.876029 35784368 PMC9243262

[36] Feng TS Zhao XM Gu P Yang W Wang CC Guo QY. Adipocyte-derived lactate is a signalling metabolite that potentiates adipose macrophage inflammation via targeting PHD2. Nat Commun (2022) 13(1). doi: 10.1038/s41467-022-32871-3 PMC9445001 36064857

[37] Wagner M Samdal Steinskog ES Wiig H. Adipose tissue macrophages: the inflammatory link between obesity and cancer? Expert Opin Ther Targets (2015) 19(4):527–38. doi: 10.1517/14728222.2014.991311 25474374

[38] Wagner M Bjerkvig R Wiig H Melero-Martin JM Lin RZ Klagsbrun M. Inflamed tumor-associated adipose tissue is a depot for macrophages that stimulate tumor growth and angiogenesis. Angiogenesis (2012) 15(3):481–95. doi: 10.1007/s10456-012-9276-y PMC3619408 22614697

[39] Wagner M Bjerkvig R Wiig H Dudley AC. Loss of adipocyte specification and necrosis augment tumor-associated inflammation. Adipocyte (2013) 2(3):176–83. doi: 10.4161/adip.24472 PMC3756107 23991365

[40] Wagner M Dudley AC. A three-party alliance in solid tumors Adipocytes, macrophages and vascular endothelial cells. Adipocyte (2013) 2(2):67–73. doi: 10.4161/adip.23016 23805401 PMC3661111

[41] Wagner M Ealey KN Tetsu H Kiniwa T Motomura Y Moro K. Tumor-derived lactic acid contributes to the paucity of intratumoral ILC2s. Cell Rep (2020) 30(8):2743–57 e5. doi: 10.1016/j.celrep.2020.01.103 32101749

[42] Howard E Hurrell BP Helou DG Quach C Painter JD Shafiei-Jahani P. PD-1 blockade on tumor microenvironment-resident ILC2s promotes TNF-alpha production and restricts progression of metastatic melanoma. Front Immunol (2021) 12:733136. doi: 10.3389/fimmu.2021.733136 34531874 PMC8438316

[43] Jacquelot N Seillet C Wang M Pizzolla A Liao Y Hediyeh-Zadeh S. Blockade of the co-inhibitory molecule PD-1 unleashes ILC2-dependent antitumor immunity in melanoma. Nat Immunol (2021) 22(7):851–64. doi: 10.1038/s41590-021-00943-z PMC7611091 34099918

[44] Kumagai S Koyama S Itahashi K Tanegashima T Lin YT Togashi Y. Lactic acid promotes PD-1 expression in regulatory T cells in highly glycolytic tumor microenvironments. Cancer Cell (2022) 40(2):201–18 e9. doi: 10.1016/j.ccell.2022.01.001 35090594

[45] Lim JX Lai CY Mallett GE McDonald D Hulme G Laba S. Programmed cell death-1 receptor-mediated regulation of Tbet(+)NK1.1(-) innate lymphoid cells within the tumor microenvironment. Proc Natl Acad Sci USA (2023) 120(18):e2216587120. doi: 10.1073/pnas.2216587120 37098069 PMC10161089

[46] Kim J DeBerardinis RJ. Mechanisms and implications of metabolic heterogeneity in cancer. Cell Metab (2019) 30(3):434–46. doi: 10.1016/j.cmet.2019.08.013 PMC6730674 31484055

[47] Pavlides S Whitaker-Menezes D Castello-Cros R Flomenberg N Witkiewicz AK Frank PG. The reverse Warburg effect: aerobic glycolysis in cancer associated fibroblasts and the tumor stroma. Cell Cycle (2009) 8(23):3984–4001. doi: 10.4161/cc.8.23.10238 19923890

[48] Wang ZH Peng WB Zhang P Yang XP Zhou Q. Lactate in the tumour microenvironment: From immune modulation to therapy. EBioMedicine (2021) 73:103627. doi: 10.1016/j.ebiom.2021.103627 34656878 PMC8524104

[49] Diem S Kasenda B Spain L Martin-Liberal J Marconcini R Gore M. Serum lactate dehydrogenase as an early marker for outcome in patients treated with anti-PD-1 therapy in metastatic melanoma. Br J Cancer (2016) 114(3):256–61. doi: 10.1038/bjc.2015.467 PMC4742588 26794281

[50] Wagner NB Forschner A Leiter U Garbe C Eigentler TK. S100B and LDH as early prognostic markers for response and overall survival in melanoma patients treated with anti-PD-1 or combined anti-PD-1 plus anti-CTLA-4 antibodies. Br J Cancer (2018) 119(3):339–46. doi: 10.1038/s41416-018-0167-x PMC6070917 29950611

[51] De Luca R Meraviglia S Blasi L Maiorana A Cicero G. Nivolumab in metastatic melanoma: good efficacy and tolerability in elderly patients. Curr Oncol (2020) 27(2):e75–80. doi: 10.3747/co.27.5293 PMC7253741 32489255

[52] Zdralevic M Brand A Di Ianni L Dettmer K Reinders J Singer K. Double genetic disruption of lactate dehydrogenases A and B is required to ablate the "Warburg effect" restricting tumor growth to oxidative metabolism. J Biol Chem (2018) 293(41):15947–61. doi: 10.1074/jbc.RA118.004180 PMC6187639 30158244

[53] Markert CL Moller F. Multiple forms of enzymes: tissue, ontogenetic, and species specific patterns. Proc Natl Acad Sci USA (1959) 45(5):753–63. doi: 10.1073/pnas.45.5.753 PMC222630 16590440

[54] Kim EY Chung TW Han CW Park SY Park KH Jang SB. A novel lactate dehydrogenase inhibitor, 1-(Phenylseleno)-4-(Trifluoromethyl) benzene, suppresses tumor growth through apoptotic cell death. Sci Rep (2019) 9(1):3969. doi: 10.1038/s41598-019-40617-3 30850682 PMC6408513

[55] Bok R Lee J Sriram R Keshari K Sukumar S Daneshmandi S. The role of lactate metabolism in prostate cancer progression and metastases revealed by dual-agent hyperpolarized (13)C MRSI. Cancers (Basel) (2019) 11(2). doi: 10.3390/cancers11020257 PMC6406929 30813322

[56] Valvona CJ Fillmore HL. Oxamate, but not selective targeting of LDH-A, inhibits medulloblastoma cell glycolysis, growth and motility. Brain Sci (2018) 8(4). doi: 10.3390/brainsci8040056 PMC5924392 29601482

[57] Xie H Hanai J Ren JG Kats L Burgess K Bhargava P. Targeting lactate dehydrogenase–a inhibits tumorigenesis and tumor progression in mouse models of lung cancer and impacts tumor-initiating cells. Cell Metab (2014) 19(5):795–809. doi: 10.1016/j.cmet.2014.03.003 24726384 PMC4096909

[58] Wu H Wang Y Ying M Jin C Li J Hu X. Lactate dehydrogenases amplify reactive oxygen species in cancer cells in response to oxidative stimuli. Signal Transduct Target Ther (2021) 6(1):242. doi: 10.1038/s41392-021-00595-3 34176927 PMC8236487

[59] He R Zang J Zhao Y Liu Y Ruan S Zheng X. Nanofactory for metabolic and chemodynamic therapy: pro-tumor lactate trapping and anti-tumor ROS transition. J Nanobiotechnol (2021) 19(1):426. doi: 10.1186/s12951-021-01169-9 PMC8684183 34922541

[60] Dikshit A Lu J Ford AE Degan S Jin YJ Sun H. Potential utility of synthetic D-lactate polymers in skin cancer. JID Innov (2021) 1(3):100043. doi: 10.1016/j.xjidi.2021.100043 34909738 PMC8659406

[61] Mayeur C Gratadoux JJ Bridonneau C Chegdani F Larroque B Kapel N. Faecal D/L lactate ratio is a metabolic signature of microbiota imbalance in patients with short bowel syndrome. PloS One (2013) 8(1). doi: 10.1371/journal.pone.0054335 PMC3553129 23372709

[62] Goldberg JS. Stereocomplexes Formed From Select Oligomers of Polymer d-lactic Acid (PDLA) and l-lactate May Inhibit Growth of Cancer Cells and Help Diagnose Aggressive Cancers-Applications of the Warburg Effect. Perspect Medicin Chem (2011) 5:1–10. doi: 10.4137/PMC.S6229 21487535 PMC3072648

[63] Piasentin N Milotti E Chignola R. The control of acidity in tumor cells: a biophysical model. Sci Rep (2020) 10(1):13613. doi: 10.1038/s41598-020-70396-1 32788634 PMC7423962

[64] Pinheiro C Miranda-Goncalves V Longatto-Filho A Vicente AL Berardinelli GN Scapulatempo-Neto C. The metabolic microenvironment of melanomas: Prognostic value of MCT1 and MCT4. Cell Cycle (2016) 15(11):1462–70. doi: 10.1080/15384101.2016.1175258 PMC4934068 27105345

[65] Tasdogan A Faubert B Ramesh V Ubellacker JM Shen B Solmonson A. Metabolic heterogeneity confers differences in melanoma metastatic potential. Nature (2020) 577(7788):115–20. doi: 10.1038/s41586-019-1847-2 PMC6930341 31853067

[66] Qu Y Dou B Tan H Feng Y Wang N Wang D. Tumor microenvironment-driven non-cell-autonomous resistance to antineoplastic treatment. Mol Cancer (2019) 18(1):69. doi: 10.1186/s12943-019-0992-4 30927928 PMC6441162

[67] Singh S Lomelino CL Mboge MY Frost SC McKenna R. Cancer drug development of carbonic anhydrase inhibitors beyond the active site. Molecules (2018) 23(5). doi: 10.3390/molecules23051045 PMC6099549 29710858

[68] Whitton B Okamoto H Packham G Crabb SJ. Vacuolar ATPase as a potential therapeutic target and mediator of treatment resistance in cancer. Cancer Med (2018) 7(8):3800–11. doi: 10.1002/cam4.1594 PMC6089187 29926527

[69] Andreucci E Peppicelli S Carta F Brisotto G Biscontin E Ruzzolini J. Carbonic anhydrase IX inhibition affects viability of cancer cells adapted to extracellular acidosis. J Mol Med (Berl) (2017) 95(12):1341–53. doi: 10.1007/s00109-017-1590-9 28929255

[70] Chafe SC McDonald PC Saberi S Nemirovsky O Venkateswaran G Burugu S. Targeting hypoxia-induced carbonic anhydrase IX enhances immune-checkpoint blockade locally and systemically. Cancer Immunol Res (2019) 7(7):1064–78. doi: 10.1158/2326-6066.CIR-18-0657 31088846

[71] Martins BX Arruda RF Costa GA Jerdy H de Souza SB Santos JM. Myrtenal-induced V-ATPase inhibition - A toxicity mechanism behind tumor cell death and suppressed migration and invasion in melanoma. Biochim Biophys Acta Gen Subj. (2019) 1863(1):1–12. doi: 10.1016/j.bbagen.2018.09.006 30279148

[72] Azzarito T Lugini L Spugnini EP Canese R Gugliotta A Fidanza S. Effect of modified alkaline supplementation on syngenic melanoma growth in CB57/BL mice. PloS One (2016) 11(7):e0159763. doi: 10.1371/journal.pone.0159763 27447181 PMC4957829

[73] Li X Yang Y Zhang B Lin X Fu X An Y. Lactate metabolism in human health and disease. Signal Transduct Target Ther (2022) 7(1):305. doi: 10.1038/s41392-021-00847-2 36050306 PMC9434547

